# Hospital and Institutional News

**Published:** 1918-11-23

**Authors:** 


### HOSPITAL AND INSTITUTIONAL NEWS.

### THE KING IN NORTH LONDON.

In the course of the Royal drive through North London on Nov. 15 two little patients of the Great Northern Central Hospital presented a bouquet of carnations to the Queen when Their Majesties' carriage paused in front of the hospital. The children were carried by nurses, and the presentation evoked hearty enthusiasm from the crowds and from the hospital staff and patients, many of whom were grouped on the pavement or the steps of the hospital, or at the windows and on the balconies.

### PEEL HALL AS A SANATORIUM.

The Lancashire County Committee has decided to convert Peel Hall, Little Hulton, into a sanatorium for tuberculosis. The county architect has prepared plans to adapt the house, at a cost of £11,000. A further £2,000 will probably be required for its equipment. If the Local Government Board approves, the County Committee will make a grant of £13,000, in return for which it expects to possess a sanatorium for forty more patients, together with the necessary staff.

### PREPARING FOR CHRISTMAS.

Mr. R. J. Coles, who proved himself a good financial organiser before he became superintendent of King's College Hospital, is now starting a special fund to meet the cost of Christmas festivities on the lines of that which proved successful at his former hospital, Addenbrooke's, Cambridge. He also appeals to Londoners for gifts in kind, as he used to appeal to the various districts round Cambridge. It used to be said that different qualities were required for successful administrators in the provinces from those which are necessary in London. Mr. Coles, we are happy to think, is making the best of both worlds.

### HURST'S SUNSHINE HOME HOSPITAL.

The Sunshine Home Auxiliary Hospital, at Hurst, Sussex, was recently brought under special notice by the visit of Princess Alice, Countess of Athlone, for the purpose of opening the newly erected Recreation Hut of St. Michael. The presence at the ceremony of some of the leading doctors from the great 2nd Eastern General Hospital, at Brighton, was a tribute to the confidence the medical profession place in this little hospital of thirty-six beds, so ably managed by a lady known as Sister Maud, whose energies are not impaired by the fact that she discharges her duties in a wheeled chair.

### WARDS FOR PNEUMONIA CASES.

A pneumonia ward has been opened at King Edward VII.'s Hospital, Cardiff, by the employment of the "Elize Nixon" flat. This contains nineteen beds for men and sixteen for women, and fourteen cases were admitted at once. Medical practitioners were warned that the beds are intended for pneumonia cases only. Influenza which has not been followed by pneumonia is an insufficient claim, and doctors have been advised, to avoid delay, to communicate at once with the resident surgical officer. The problem was brought home to the hospital not only by the epidemic, but from the fact that twenty-seven of its own nurses were attacked, fortunately without complications. The example at Cardiff deserves consideration elsewhere.

### LACK OF NURSES AT BIRKENHEAD.

On account of the large amount of sickness prevailing in Birkenhead and district and the difficulty in securing nurses the Birkenhead Guardians have appealed for offers of service (voluntary or otherwise) from educated women for whole- or part-time service at the Tranmere Military and Civil Hospital, to assist the present nursing staff. The Guardians have also decided, as an emergency measure, to suspend the visiting of patients by relatives and friends unless the patient is on the danger list. By this means it is hoped the spread of the disease will be checked. Doctors and chemists have been overwhelmed with work in the district, and in several cases have themselves fallen victims to influenza.

### BRIGHTON WOMEN'S HOSPITAL NEEDS HELP.

Despite the fact that an attractive little poster advertised in the towns of Brighton and Hove Pound Day in connection with the Brighton Women's Hospital, and the earnest appeal from Lady Leconfield, the President, there was not that response that the institution hoped for and deserved. Owing to the condition of the country consequent upon the war there are few institutions that can be regarded as of more importance than those which specialise in maternity. An A1 empire cannot be run by a C3 population, as Mr. Lloyd George said the other day. The hall-marks of the Brighton institution are its eighty-eight years' existence and its proud record of 1,000 births per annum, equal to a third of the maternity cases of the local population. Furthermore, it is the only maternity hospital in Sussex.

### INSTITUTIONAL FARMING.

The York City Asylum has carried on a quiet but successful year, especially, perhaps, in regard to its farm, notwithstanding controlled prices of various produce and stock, and the high cost of cake, meal, and, indeed, of animals. The price of milch cows has been, of course, high. At the dairy farm labour has been saved by the adoption of a mechanical milker, while the yield of barley, oats, and wheat was satisfactory. The potato crop suffered in consequence of the dry weather in May and June, but turnips did well, and also mangolds. To farm at a profit when "feeds" are inferior in quality and high in price, and when the price of stock has been controlled, is difficult; but the main object of an institutional farm is rather to save expense and to supply the institution with food and the fit patients with employment than to bear much of the general cost of maintenance.

### DEATH OF DR. JOHN McCAW.

SINCERE regret was expressed at the Lambeth Infirmary at the death from influenzal pneumonia of Dr. John McCaw, F.R.C.S., M.D., who for the past three years has been a temporary assistant medical officer. He was only thirty-three years of age, and gave promise of a brilliant career. Dr. Lionel Baly, the medical superintendent, in reporting the sad loss to the Lambeth Board of Guardians, stated that he possessed exceptional abilities, having gained the degrees of F.R.C.S. and M.D. (Edinburgh) last year. His professional prospects were undoubtedly excellent, and it was his intention at the close of the war to return home to New Zealand, where his parents reside. His brothers, Captain Alexander McCaw and Captain Cuthbert McCaw, both of the R.A.M.C., New Zealand, were able to be present at his funeral.

### THE RARER TYPE OF CHAIRMAN.

MANY hospital chairmen take little overt interest in the life during training of their nurses. But there are some who are not content to occupy themselves with finance, appointments, and administration, but attempt to become the father and friend of the nursing staff. Since the voluntary system is, of its proper principle, personal rather than official in character, such attempts are always interesting, and their motive is praiseworthy. But this attempt requires much tact and time to carry out with success. It means that the chairman will appear at any time, by night as well as day, for the night staff will interest him no less than the day nurses. We know of some, of one or two, who appear at all hours, chat, observe, even advise—a type of man as quick to note the comfort or need of a patient in small things as to look to those of the nurses. A man of long experience will gather a surprisingly long list of nursing "tips" which will startle and arrest a young probationer, and, if he has the art of speaking, can deliver more or less informal addresses to the nurses which can make them realise that an attentive observer can see, in the course of years, a great deal of "the game."

### THE NURSES' FATHER CONFESSOR.

NATURALLY, a chairman who lives in his hospital to this extent must become a subject of much discussion to the nursing staff, the junior members of which will be prone either to idolise or to dread him. He will make himself accessible to everyone at all hours, and the youngest probationer can bring, if she chooses, to his personal notice, the most trivial grievance, or, possibly, an intimate personal confession. He will become, if he proceeds with judgment, the father confessor to the whole institution, which will become imbued with his spirit for good or ill. Such a man is bound to make mistakes; but a man of such lively devotion to his hospital will be capable of learning from them. His devotion of time and attention cannot but make him an autocrat, a benevolent despot, shall we say? Few have the time for such a life in the wards. But a few have attempted it with considerable measure of success. Is this type of chairman generally approved; or, because few have the time for its demands, should we be glad that such chairmen are exceptional?

### BERMONDSEY GUARDIANS CENSURED.

THE Local Government Board have issued their report on the inquiry held by Mr. J. S. Oxley, their inspector, into the large purchases of drapery by the clerk to the Bermondsey Board of Guardians (Mr. E. Pitts Fenton), to which reference was made in the columns of THE HOSPITAL on July 20, page 339. The report is a most drastic condemnation of both the Guardians and the clerk. The Board state that they cannot acquit the Guardians of a laxity of administration which was inconsistent with their obligations to the ratepayers. If the Guardians had not failed, both as a body and individually, to acquaint themselves with the matters placed before them for approval, and if, in particular, the documents relating to the purchases in question had been examined with due care by the members who took the responsibility of authenticating them by their signatures, the purchases which were being made by the clerk would have been checked in time, and the occasion for holding the inquiry would not have arisen.

### THE LIABILITY OF THE CLERK.

MR. OXLEY states that the effect of his conclusions was that though the clerk was not proved to have been guilty of any criminal practices, he placed on the resolution of the Guardians empowering him, in conjunction with the chairman of the Contracts Committee, to make purchases an interpretation which he knew was beyond the intentions of the Guardians when they passed it; and he relied on the casual way in which the ordinary work of the Guardians was done as being likely to ensure that he would not be interfered with until he had completed the purchases of textiles he contemplated making. It would not have been possible for the clerk to have made these purchases without the knowledge of the Guardians if they had taken a reasonable interest in their ordinary duties. The Local Government Board, in agreeing with Mr. Oxley, add, at the same time, they were unable to hold that the clerk had properly discharged his duties as clerk to a public authority. In their opinion, he took advantage of the unbusinesslike methods of the Guardians to carry through a series of transactions which, though he may have believed them to have been in the best interests of the Poor-Law Union, he must have known to be contrary to the Guardians' policy and opposed to the spirit of the Local Government Board's letter in which they sanctioned the departure from the regulations empowering the purchases to be made without advertising in the usual way for tenders.

### THE GUARDIANS' ATTITUDE.

THE wholesale condemnation of their laxity naturally did not meet with the approval of the Bermondsey Board of Guardians, as they knew that as soon as they discovered the purchases had been made they took immediate steps to stop them, although their intervention came too late. The work of the Guardians since the commencement of the war has been done by ten or twelve members out of twenty-four, some of the absentees being on active service, whilst the majority have failed to attend either the meetings of the Board or the committees. They accordingly adopted a resolution protesting against the strictures passed upon them by the Local Government Board, and expressing the opinion that the clerk had committed an error of judgment, but, in view of his services for over twenty-seven years, they decided to take no further action. This was carried by nine votes to eight, the minority desiring that the Local Government Board should be asked to suspend the clerk from his duties for grave misconduct. There the matter ends for the present, but an attempt is to be made to rescind the resolution carried at the last meeting. With regard to the valuation of the drapery, the experts appointed by the Local Government Board report that goods costing £11,509 bought by the clerk had risen in value to £18,178. This, however, is entirely outside the important point involved in the inquiry as to whether a public authority should purchase large stocks for which they have no immediate use.

### THE CITY GUARDIANS AND ST. BARTHOLOMEW'S.

A SERIOUS dispute has arisen between the City of London Board of Guardians and the Governors of St. Bartholomew's Hospital. The Guardians have been in the habit of subscribing £31 10s. annually to the hospital funds, and when this came before them for annual renewal it was pointed out that a man suffering from nephritis, had been sent to Thavies Inn from the hospital as they were unable to admit him. He died soon after admission. They felt that, considering his very serious condition, special arrangements should have been made for his reception at the hospital, and that his transfer to the infirmary hastened his death. It was maintained that there were other complaints almost of a similar character, and under the circumstances they decided to withhold their annual subscription. Lord Sandhurst, in a letter to the Guardians, said he recognised that this decision was intended as a public indication of want of confidence in the management of the hospital, and, in the circumstances, he had no alternative but to request that his application for a renewal of the subscription which the Guardians had made annually should be withdrawn. There the matter ends for the present.

### PEACE APPEALS.

ON the morning following Peace Day, or rather the day of the signing of the Armistice, a large number of Londoners received an appeal from Sir Arthur Pearson on behalf of St. Dunstan's Hostel for Blinded Soldiers. Although Sir Arthur was not alone in the idea of a peace appeal, he was the first in the field, and, not being restrained as to expense by any controlling central funds, such as are the voluntary hospitals, he was able to make his effort on a big scale, and we wish it a big success. Six hospitals later in the week stepped with a joint appeal into the arena, and issued 100,000 letters, the cost of which had been met by private subscription. These institutions are the East London Hospital for Children, the Chelsea Hospital for Women, King's College Hospital, the City of London Hospital for Diseases of the Chest, the National Hospital for the Paralysed and Epileptic, and the British Hospital and Home for Incurables. This collective effort is managed by Mr. J. S. Wood, the Editor of the *Gentlewoman*, himself an ex-secretary and a gentleman who has had success in the money-raising way. The Metropolitan Hospital asks for 200,000 florins, the Hospital for Epilepsy and Paralysis and Nervous Diseases asks for a financial token of the relief of nervous tension, and All Saints' Hospital, the Evelina Hospital, and some others also invite peace offerings. The voluntary hospitals have been so modest in their demands during the war that now the burdens of the charitable are somewhat lightened by the surcease of some war charities, we hope that these older institutions may receive more notice and support.

### WEAKNESSES OF WELFARE WORK.

DR. ELSPET BURSEY, the Durham County Welfare Medical Officer, criticises some of the maternity and child-welfare centres in his latest quarterly report. At "Babies' Welcomes" at Durham and Leadgate there was frequently no doctor in attendance, although one of the main inducements to expectant mothers to attend is the chance of timely advice. Only a doctor with his or her medical training can examine or usefully advise, for the health visitor's advice is necessarily limited, and not comprehensive. Dr. Bursey's dictum is that "Babies' Welcomes" without a doctor in attendance are practically worthless. At two centres, where there is no separate doctor's room, no ante-natal work can be done, and this is, he says, emphatically the most important branch of the work. At some centres, notably Leadgate, there appears to be little effort among the voluntary workers to specialise in baby clothing. This, the report points out, is essentially the province of the voluntary woman worker. Lastly, larger and more detailed cards are necessary accurately to record the sociological conditions as well as the medical facts of each case. Only co-operation with the medical officer in this matter of record cards can secure the uniformity in figures and facts necessary for their future statistical collation.

### THE FINANCE OF TRANSITION.

THE immense problems resulting from peace, for so we may venture to call it, will probably prove more full of difficulty for the voluntary hospitals than the emergency created by the war. That provided an external pressure, a sense of tangible danger, which was apparent to all. Will the nation rise to the no less real, but less superficially apparent, demands of a return to peace and civil order? The problem can best be seen in a concrete case. The Birmingham General Hospital finds that the average cost of treating an in-patient is close on £8. It finds, too, that so long as present high prices last, an additional income of £15,000 a year will be necessary. The sum total of the debt at the end of the year is expected to be £37,000. The Saturday Fund at Birmingham and elsewhere is trying to increase, or at least to maintain the recent increase in its contributions. The rate per head, which has been raised before, may possibly be raised again. But the Saturday Fund cannot be expected to shoulder the whole burden. It should not be asked to do so. Have the employers been as generous in proportion as their workpeople? Or does the casual cheque still suffice? The scheme which Dr. Basil Rhodes, before his departure for work in London, arranged among the employers and other sections of the community in North Staffordshire, can be applied to Birmingham. Its essence is a co-operative and friendly effort, voluntarily severally applied to themselves by both employers and employed.

### POST-GRADUATION STUDY AT GLASGOW.

A recent meeting of the Glasgow University Court shows that the educational authorities there are alive to one aspect of the medical situation which will arrive with the return of some thousands of doctors from service in the Army to civilian practice. Many of these are young men who stepped straight from a student training into Army work, and still more have been out of touch with the routine of family practice, or perhaps even engaged in purely administrative duties. Without question there will be a considerable demand from these and other sources for opportunities for clinical and laboratory study prior to a return to family practice. Clearly it is the duty of the medical schools to see that this demand is met, and no time is to be lost if the arrangements are to be well and thoroughly organised. The question was raised in the Glasgow University Court by Dr. Geo. S. Middleton, who was elected some few months ago as one of the representatives on the Court of the graduates, and there will be widespread approval of his action. It was decided to ask the Medical Faculty to prepare a detailed plan, and it may be hoped that the matter will be pressed forward with all possible speed. Further, the example set in the North ought to be followed elsewhere, and indeed the whole question of post-graduation medical teaching needs the prompt application of practical measures. Not only our own practitioners have to be considered, but we ought also to provide for students from the whole of the English-speaking world. Berlin and Vienna used to attract these, as here there was no efficient organisation to meet their wants. But the Central European capitals have lost their place of pride, and it falls to Great Britain to take up the task.

### ENGINEERS AND DISEASE PREVENTION.

Under this title a sanitary engineer makes an appeal in *The Times* for a more definite status to be given to his profession in reference alike to municipal and to domestic sanitation. His claim is that even when schemes are arranged by the expert the execution of them is very often left in incapable hands, and further that plans well founded and well carried out need continuous and competent supervision. A false economy endeavours invariably to minimise expense, even though efficiency suffers in the process, and, as much of the work is out of sight, it fails to command appreciation or to receive capable supervision. Hence even good schemes become inefficient, and mischiefs duly follow. The position in these respects is better than it used to be, but it still leaves much to be desired. We agree that more use ought to be made of the well-trained and expert sanitary engineer in the arrangement and control of drainage and kindred schemes. Nowadays medicine is keen, and rightly keen, on what may be called specific prevention, that is the removal of the bacteriological agents which are known to be the causes of specific disease. But it still remains true that "dirt" has dangers of its own, and that it often supplies the opportunity for pathogenic organisms to multiply. Hence the standard of general cleanliness ought to be maintained, or, still better, raised, and the sanitary engineer has a particular function to this end. One hindrance, we think, is the want of a precise definition, for it would seem as if the plumber of yesterday may be free to-morrow to take on the more stately designation.

### THIS WEEK'S DRUG MARKET.

The drop in drug prices is slow in coming, but there can be no doubt that it will come; let there be no mistake about it. In the meantime it is remarkable how few drugs are actually quoted at lower prices. Nevertheless, almost everything is, to use a market expression "on the easy side." The fact is that the market has not been thoroughly tested since the signing of the Armistice, for nobody is buying what he can do without, while holders, anxious to put on as bold a front as possible, are not offering any bargains. It may be weeks, it may be months, but prices are bound to come down. Therefore hospital buyers should not buy more than they want to keep them going from day to day or week to week. One thing is certain: the end of the war cannot make drugs dearer.

### TO OUR READERS.

Contributions are specially invited from any of our readers to these columns. They should deal with topical subjects and news. They must be authenticated for the information of the Editor only. The minimum payment if published is 5s. There is no hard-and-fast rule as to space, but notes of about twenty lines in length are preferred.

